# Macroevolution along developmental lines of least resistance in fly wings

**DOI:** 10.1038/s41559-025-02639-1

**Published:** 2025-02-07

**Authors:** Patrick T. Rohner, David Berger

**Affiliations:** 1 Department of Ecology, Behavior, and Evolution, University of California, San Diego, La Jolla, CA USA; 2 Department of Ecology and Genetics, Uppsala University, Uppsala, Sweden

**Keywords:** Evolutionary genetics, Evolutionary developmental biology

## Abstract

Evolutionary change requires genetic variation, and a reigning paradigm in biology is that rates of microevolution can be predicted from estimates of available genetic variation within populations. However, the accuracy of such predictions should decay on longer evolutionary timescales, as the influence of genetic constraints diminishes. Here we show that intrinsic developmental variability and standing genetic variation in wing shape in two distantly related flies, *Drosophila melanogaster* and *Sepsis punctum*, are aligned and predict deep divergence in the dipteran phylogeny, spanning >900 taxa and 185 million years. This alignment cannot be easily explained by constraint hypotheses unless most of the quantified standing genetic variation is associated with deleterious side effects and is effectively unusable for evolution. However, phenotyping of 71 genetic lines of *S. punctum* revealed no covariation between wing shape and fitness, lending no support to this hypothesis. We also find little evidence for genetic constraints on the pace of wing shape evolution along the dipteran phylogeny. Instead, correlational selection related to allometric scaling, simultaneously shaping developmental variability and deep divergence in fly wings, emerges as a potential explanation for the observed alignment. This suggests that pervasive natural selection has the potential to shape developmental architectures of some morphological characters such that their intrinsic variability predicts their long-term evolution.

## Abstract (web summary)

Analysing wing shapes within and across distantly related dipteran lineages, the authors show that intrapopulation variation reflecting microevolutionary change can predict deep divergence at macroevolutionary timescales spanning 185 million years, and suggest that this pattern can be explained by correlational selection related to allometric scaling.

## Main

A central aim in evolutionary biology is to predict evolution. Quantitative genetic approaches have played an important role in this endeavour by leveraging within-population estimates of evolvability in the form of standing genetic variation and de novo mutational variation in quantitative traits to predict their evolution^1–4^. The translation of mutational variation at the nucleotide level into variation at the level of the phenotype is governed by developmental processes. Frequently, these processes channel random nucleotide changes into non-random phenotypic variation, giving rise to developmental bias^5–7^. These biases are recognized to impact future adaptation by generating abundant substrate for evolution along certain phenotypic dimensions, while limiting it in others^5,6,8–11^. However, much controversy surrounds the timescale on which these biases constrain evolution. In particular, while limited genetic variation is predicted to slow down evolution, it is not expected to prevent phenotypic change entirely, and estimates of evolvability within single populations are therefore expected to be poor predictors of macroevolutionary diversification^12–14^.

Yet, several recent studies have challenged this standard expectation by showing correlations between evolvability estimates within populations and rates of macroevolution^15–18^. One line of evidence comes from studies on morphological evolution where mutational and standing genetic variation in morphological traits predict their long-term divergence^16,18–20^. Owing to the timescales over which evolution was observed, these relationships are hard to reconcile with the sole action of genetic constraints. Alternatively, it has been suggested that such correlations could result from natural selection that shapes the phenotypic effects of de novo mutations^4,6,11,21–24^. According to this hypothesis, stabilizing selection moulds development so that deleterious effects of segregating genetic variants become reduced^24,25^ while the phenotypic effects of alleles under persistent directional or fluctuating selection instead become magnified^26–28^. In this process correlational selection acts on specific trait combinations so that developmental bias evolves, with the result that fitness-reducing phenotypic outcomes of mutations may become less frequent than expected by random chance.

If past forces of selection indeed bias the phenotypic effects of de novo mutations, this would suggest that the causal relationships between the processes of mutation, selection and adaptation are more intricate than often assumed under standard models of evolution, with important implications for our ability to predict future evolution from current quantitative genetic parameters^6,22,28,29^. However, the role of selection in shaping mutational effects (that is, developmental biases) remains controversial and has been disputed on theoretical grounds^25,26,30–36^, and reconciling the observed relationships between evolvability and macroevolution with processes occurring at microevolutionary scales remains a fundamental challenge ^19,20,29,37–39^.

Here we address this controversy by extending recent analyses on the relationship between developmental bias and evolutionary divergence in dipteran wings. Houle et al.^18^ demonstrated that mutations cause non-random phenotypic variation in the wings of *Drosophila melanogaster* and, astonishingly, predict 40 million years of divergence across the Drosophilidae. These results were recently complemented by a study^16^ showing that intrinsic developmental variability in the wings of sepsid flies, a clade that diverged from the Drosophilidae around 60 million years ago, is related to the mutational variability and macroevolutionary patterns observed by Houle et al.^18^. Here we show that this alignment holds on even longer timescales, providing evidence for a relationship between de novo mutational input and macroevolution that unfolded over 185 million years. We show that the genetic constraint hypothesis alone is a poor fit to the observed patterns. Alternatively, correlational selection on wing traits as a causative agent shaping both developmental bias and deep divergence remains a plausible, yet disputed, explanation for the observed pattern. Irrespective of the ultimate explanation(s), our findings show that deep divergence in dipteran wings can be reasonably well predicted from their intrinsic developmental variability, even when such variability fails to predict evolution on shorter timescales. This challenges our understanding of the processes that govern the emergence and evolution of phenotypic variation.

## Results

Developmental bias can be assessed by studying how genetic or environmental perturbations affect developmental outputs in the form of phenotypic variation. Here we define developmental bias as the degree of anisotropy (or, reversibly, deviations from isotropy): that is, the propensity of a structure to vary more in some dimensions than in others^22^. One way of quantifying developmental bias is to study how phenotypic variation in multivariate characters is generated by de novo mutation, captured by the mutational variance–covariance matrix, *M*, an approach used by Houle et al.^18^ to capture mutational bias in wing shape in *D. melanogaster*. An alternative way of quantifying bias is to estimate the degree of variability in the developmental system by measuring fluctuating asymmetry between left and right homologues of paired bilateral structures. Because the left and right sides of the same organism share the same genome and environment, differences between bilateral homologues can be attributed to developmental noise, and differences in the degree of fluctuating asymmetry among phenotypes thus serve as a measure of bias in the developmental program^40,41^. This approach was used by Rohner and Berger^16^ to capture the developmental covariance matrix, *D*, for wing shape in sepsid flies. This covariance matrix thus captures how traits (co)vary in response to random developmental perturbations. Here we first use these previous estimates of *M* (from ref. ^18^) and *D* (from ref. ^16^) to predict 185 million years of macroevolution across 43 families and >900 species of Diptera. We then evaluate competing hypotheses invoking genetic constraints and correlational selection to reconcile the observed alignment between the generation of de novo variation and deep macroevolution.

**Fig. 1:**
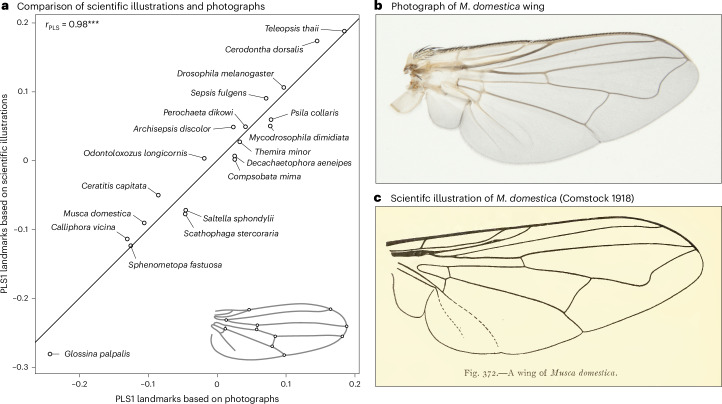
Leveraging scientific illustrations to quantify wing shape evolution. **a**, PLS plot showing a strong correlation between the shape measurements derived from photographs and those derived from scientific illustrations for 19 species where both sources of data were available. **b**,**c**, Similarity between a photograph of the wing of a house fly (*Musca domestica*) (**b**) and an illustration of a wing of the same species (**c**). Panel **c** adapted from ref. ^106^, Comstock Publishing Company. Source data

**Extended Data Fig. 1:**
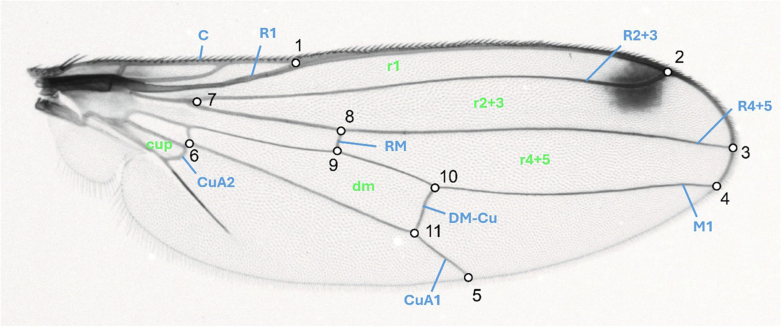
Morphology a sepsid fly wing. Location of two-dimensional landmarks used for this study illustrated on a picture of a sepsid wing. Wing veins are indicated in blue. Cells are indicated in green. Nomenclature follows [^43^ and^108^].

**Extended Data Fig. 2:**
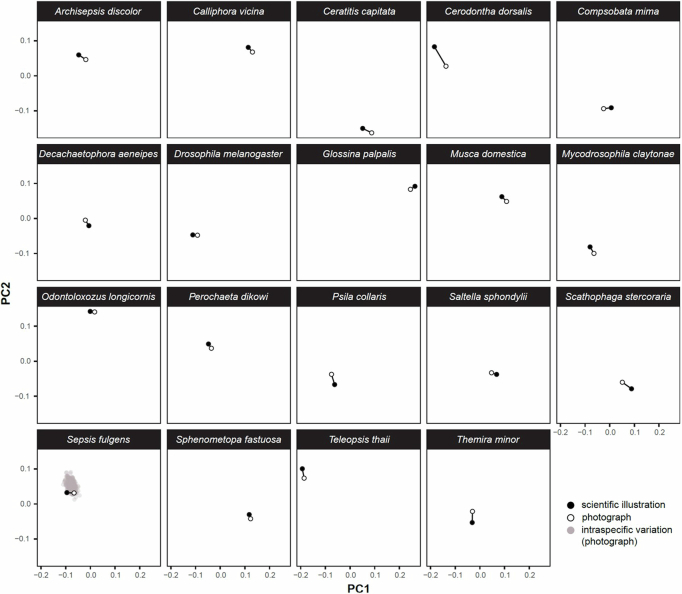
Similarity between morphological measurements sourced from scientific illustrations and pictures. Morphospace (principal components) showing the similarity between wing shape measured on photographs or scientific illustrations from the systematic and taxonomic literature. The intraspecific variation observed in *Sepsis fulgens* (data from^62^) was projected into the morphospace to provide a reference point for the amount of morphological disparity that can be expected in natural populations. Source data

We focus on the evolution of wing shape in the Eremoneura, a clade within the higher flies (Brachycera) that is about 185 million years old^42^ and contains more than 64,000 species (including, among others, the fruit, flesh, house and tsetse flies). To quantify variation in landmark positioning (Extended Data Fig. 1 and Extended Data Table 1) within and between families, we took advantage of published scientific illustrations and photographs of fly wings from the taxonomic and systematic literature (for example, refs. ^43,44^) (Fig. 1 and Supplementary Table 1). Our final dataset contained 988 observations from 933 different species. To first assess the accuracy of using illustrations (*n* = 414) compared to photographs of wings (*n* = 574), we computed the partial least-squares (PLS) correlation between coordinates derived from illustrations and those derived from photographs for 19 species where both types of data were available. This correlation was very strong and statistically significant (*r*_PLS_ = 0.98, *Z* = 4.21, *P* < 0.001; Fig. 1 and Extended Data Fig. 2). We found similarly strong associations when comparing the average wing shape for each genus based on illustrations with the average shape calculated on the basis of measurements taken from photographs (*r*_PLS_ = 0.93, *Z* = 6.35, *P* < 0.001, *n* = 64), showing that shape information derived from illustrations adequately captures shape variation derived from photographs.

### Developmental variance correlates with macroevolution

**Fig. 2:**
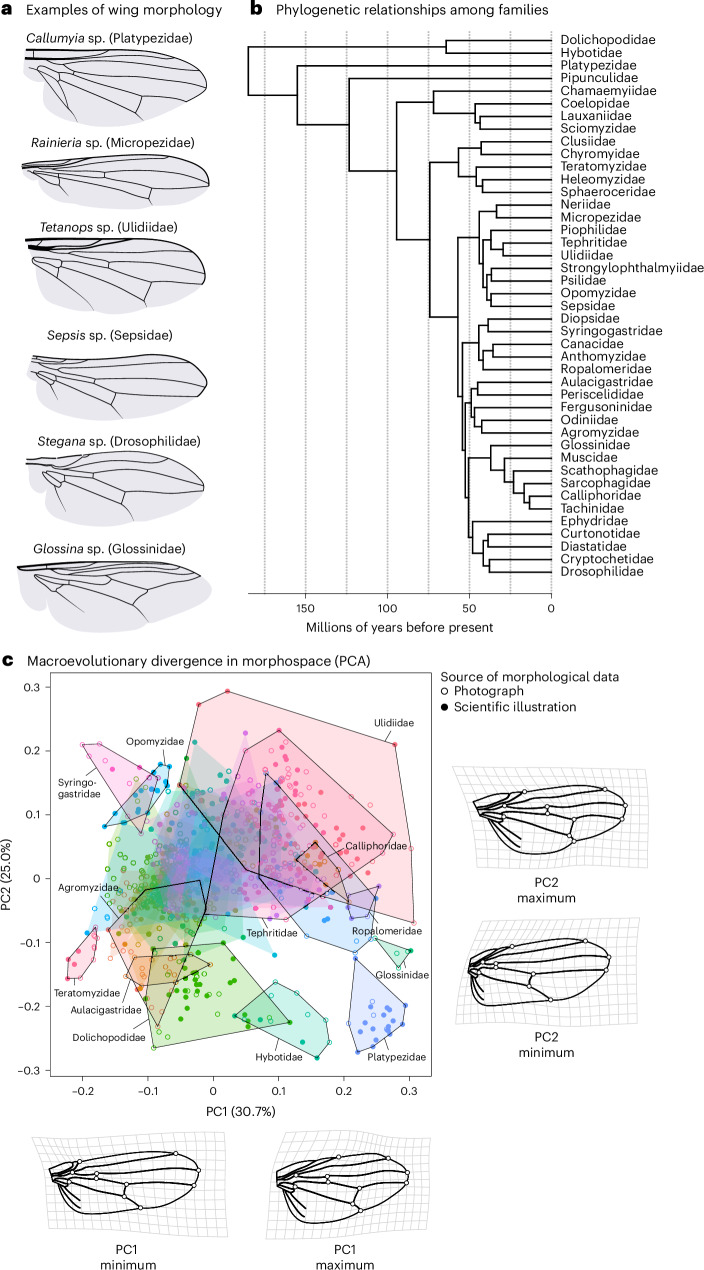
Macroevolutionary divergence in fly wing shape across 185 million years. **a**, Although fly wings evolve slowly, there is large macroevolutionary divergence in wing shape, as highlighted in these examples. **b**, Evolutionary relationships among the different families within the Eremoneura^47^. The phylogeny was calibrated using the approximate age of the Eremoneura^42^, as well as the split between Drosophilidae, Muscidae and Tephritidae^94^. **c**, An evolutionary morphospace defined by the first two principal components. Individuals are grouped by family (hulls). An arbitrary set of families is highlighted. Families with less than five observations were excluded from the plot. Shape deformations associated with minimal and maximal loadings relative to the average wing shape are indicated with deformation grids for the first two principal components. To improve visibility, the magnitude of shape changes was reduced by a factor of 0.5. The outline of the wing was based on a drawing of *Camptoprosopella vulgaris* (Lauxaniidae) after Curran^107^. PCA, principal component analysis. Source data

**Extended Data Fig. 3:**
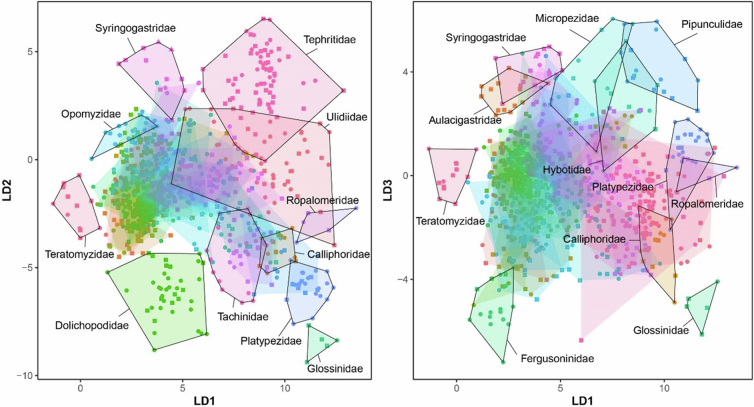
Canonical variate analysis. Evolutionary morphospace defined by the first three canonical variates. Individuals are grouped by family (hulls). An arbitrary set of families is highlighted. The source of the shape data is indicated with shape (scientific illustration and pictures). Families with less than five observations were excluded from the analysis, leading to a total number of 43 families (n = 884). The overall leave-one-out cross-validation success of the CVA was 82.3%. Source data

The 43 fly families analysed differed strongly in their wing shape (Fig. 2; Procrustes analysis of variance: *F*_42,926_ = 49.60, *Z* = 27.74, *P* < 0.001, *R*^2^ = 0.73). Leave-one-out cross-validation led to a correct classification of 82.3% of all individuals (canonical variate analysis), indicating that fly families can be differentiated on the basis of wing shape (Supplementary Table 2 and Extended Data Fig. 3). To quantify the macroevolutionary dynamics of wing shape, we computed the evolutionary rate matrix *R* on the basis of the inverse of the phylogenetic relationship matrix among dipteran families^45^ using animal models in ASReml-R (v.4.1.0.154)^46^. Because large species-level phylogenies are lacking on this broad phylogenetic scale, we based our analysis on a recent phylogeny that leveraged transcriptomes (3,145 genes) to resolve the phylogenetic placement among families^47^ (Fig. 1). The species represented in our database that fall within these families were treated as replicated measures for each family’s wing shape at the tip of the phylogeny. Macroevolutionary divergence was mostly related to the relative positioning of the first branch of the radial vein and the placement of the two cross-veins along the proximo-distal axis (Fig. 2).

Next, we tested whether deep divergence among families is related to mutational and developmental bias observed in drosophilids and sepsids. Specifically, we compared *R* to the previously estimated *D* and *M* matrices in *S. punctum* and *D. melanogaster* using a modified version of Krzanowski’s common subspace analysis following the method described previously^48^ (also see refs. ^18,49^). In brief, we compared the logarithmized variances of both matrices along the same set of orthogonal phenotypic dimensions of the wing. To limit bias in our estimates of effect sizes^48,49^, we chose to represent these phenotypic dimensions by the eigenvectors of an independently estimated third matrix—the phenotypic variance–covariance matrix, *P*—measured in *S. fulgens* (a morphologically distinct but relatively close relative of *S. punctum* placed in the same species group^50^ within the same genus). For consistency, we used this matrix as the reference to generate comparisons of different variance–covariance matrices throughout this study. However, we also repeated all comparisons using other matrices as the reference (Extended Data Table 2), which showed that our conclusions do not depend on the matrix chosen as the reference.

**Fig. 3:**
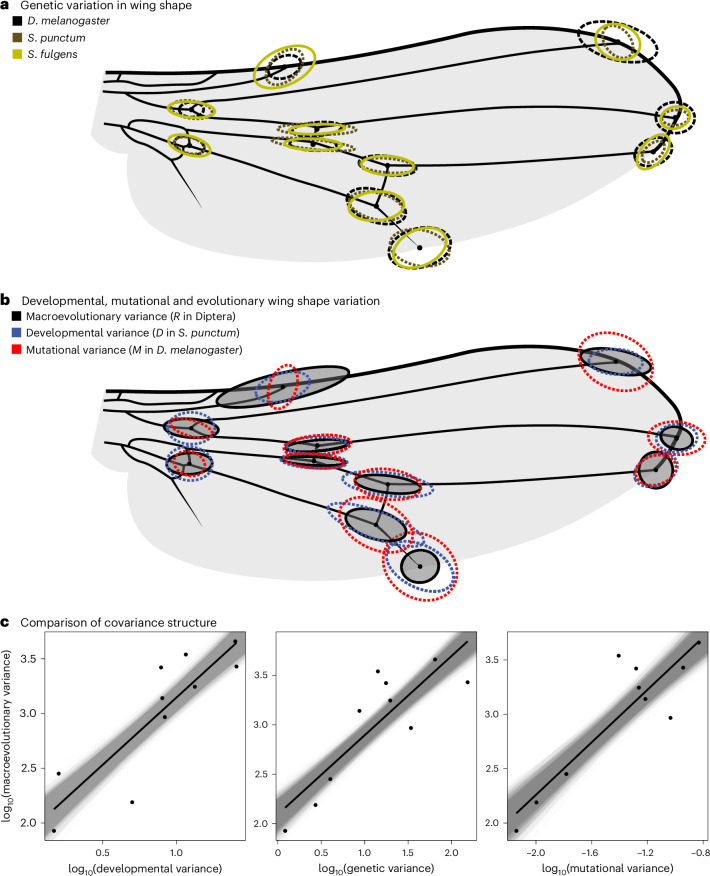
Developmental, mutational and genetic variation predict macroevolution. **a**, Similarity in standing genetic variation in wing node positioning across *Drosophila* and two sepsid flies that diverged ~64 million years ago. For the purpose of illustration, the average location of each landmark was shifted to match a typical sepsid wing. **b**, Variation in wing shape due to developmental (*D*), mutational (*M*) and macroevolutionary (*R*) variation. The matrices shown in **a** and **b** were scaled by their trace to facilitate comparison. **c**, Results of a common subspace analysis where the amount of developmental, standing genetic and mutational variance predicts the macroevolutionary variance along the same set of orthogonal vectors (that is, the first ten eigenvectors of the phenotypic variance–covariance matrix estimated in *S. fulgens*). The grey lines indicate the distribution of regression slopes using REML-MVN resampling (*n* = 10,000). Source data

**Extended Data Fig. 4:**
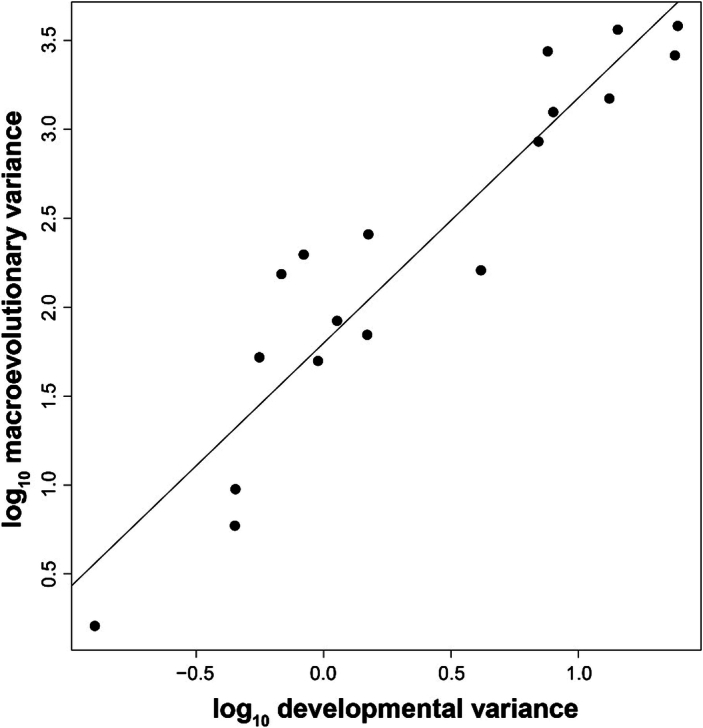
Relationship between developmental and macroevolutionary variance along all 18 eigenvectors. Results of a common subspace analysis where the amount of developmental variance predicts the macroevolutionary variance along all possible 18 eigenvectors of the **P** matrix estimated in *S. fulgens* (slope = 1.38, r = 0.94). Source data

To make sure that all matrices were compared along subspaces in which there was statistically verified variation, we estimated the rank of the matrices by using factor analytical modelling using ASReml-R^46^. The matrices were then compared along the first *k* dimensions of *P*, with *k* equal to the rank of the matrix with the lowest rank (*k* = 10). We applied this approach for any pair of variance–covariance matrices compared in this study. If macroevolutionary divergence across dipteran families can be predicted by developmental bias, we expect *R* to show similar relative amounts of variation as *D* and *M* along the eigenvectors of *P*. Regressing the resulting (logarithmized) variances of *R* on the corresponding variances of *M* and *D*, we indeed found that the morphological variation representing macroevolutionary change is similar to that generated by mutation (*R* on *M*: *β* = 0.66 (95% confidence interval, (0.53, 0.77)); *r* = 0.89 (0.78, 0.94); Fig. 3) and developmental perturbations (*R* on *D*: *β* = 0.61 (0.5, 0.71); *r* = 0.87 (0.78, 0.92); Fig. 3). When we analysed all 18 phenotypic dimensions of the wing by also including the eight additional wing dimensions for which we could not statistically certify significant variation at all biological levels compared, the relationships became even stronger (Extended Data Fig. 4). This suggests that macroevolutionary divergence among 43 dipteran families that unfolded over 185 million years is aligned with developmental lines of least resistance and can thus—at least to some degree—be predicted from intrinsic developmental variability documented in single species.

### No evidence for pleiotropic constraints on wing evolution

The relationship between developmental bias and macroevolution is consistent with fundamental constraints of wing shape development and evolution. However, owing to their polygenic basis and large mutational target sizes, the evolution of quantitative characters is typically not expected to be strongly constrained over the long time frames studied here^2,20,51^. Indeed, the study on drosophilids by Houle et al.^18^ found that a lack of mutational input is unlikely to explain the alignment between *M* and *R* in drosophilid wing evolution. To explore the possible influence of genetic constraints on the studied macroevolution of wing shape, we calculated the expected amount of divergence along the ten analysed wing shape dimensions (Fig. 3) under a scenario of pure genetic drift, which predicts that the rate of divergence should correspond to two times the mutational variance per generation^52^. Thus, if genetic constraints are limiting the evolution of some wing dimensions, we expect that the observed rates of divergence should be approximated by the predicted divergence based on the rate of mutational input. However, assuming an average of a single fly generation per year, and basing our calculations on estimates of *M* in *D. melanogaster*^18^, we found that the observed macroevolutionary variance along each of the ten dimensions is around 10^4^ times smaller than expected under drift (Extended Data Table 3). Because most species studied here undergo more than one generation per year, this calculation underestimates the expected divergence under drift (for instance, central European populations of *S. punctum* have at least four generations per year, and *D. melanogaster* has about 15 generations per year^53,54^). Thus, genetic constraints alone are unlikely to explain low rates of divergence and the observed correlation between developmental bias and macroevolution.

**Fig. 4:**
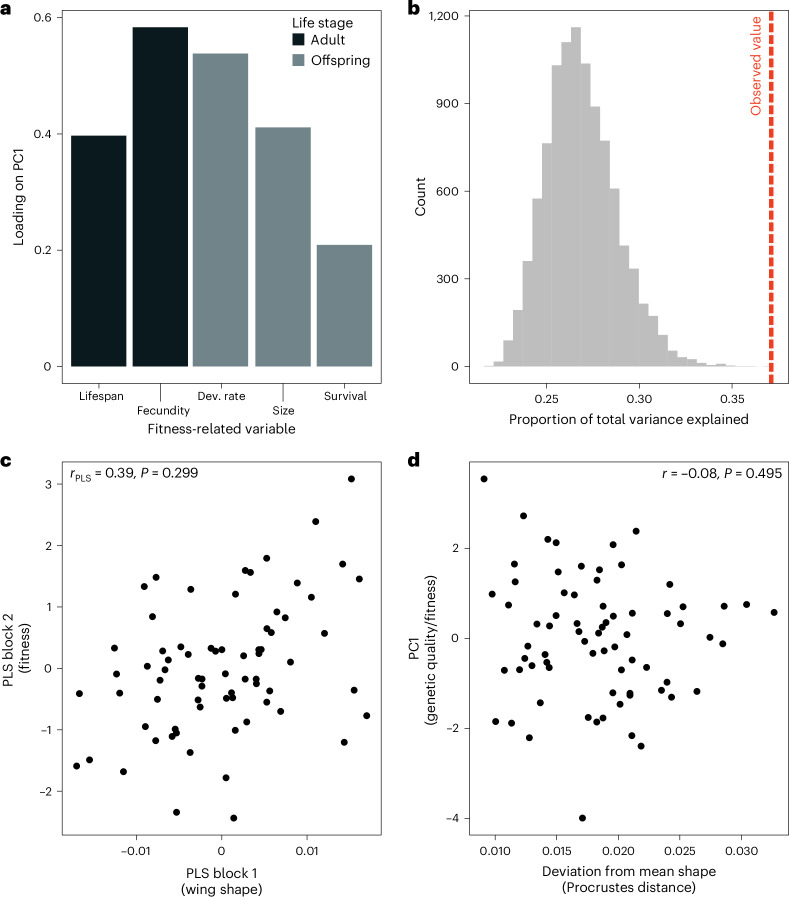
No evidence for deleterious pleiotropy constraining wing shape evolution. **a**, Loadings of fitness components measured in adults and offspring on PC1 all have the same sign, suggesting that different fitness components are correlated, which indicates a main axis of genetic quality. **b**, PC1 explained more variation than expected by chance (the red dashed line indicates the observed variance; the grey bars indicate the null distribution). **c**, Despite significant genetic variation in wing shape and fitness, we found no relationship between wing shape and the five fitness components investigated (two-block PLS analysis). **d**, There was also no relationship between the isofemale lines’ fitness (here indicated as scores on PC1) and their multivariate distance to the average wing shape, as expected if wing shape was under stabilizing selection. Source data

**Extended Data Fig. 5:**
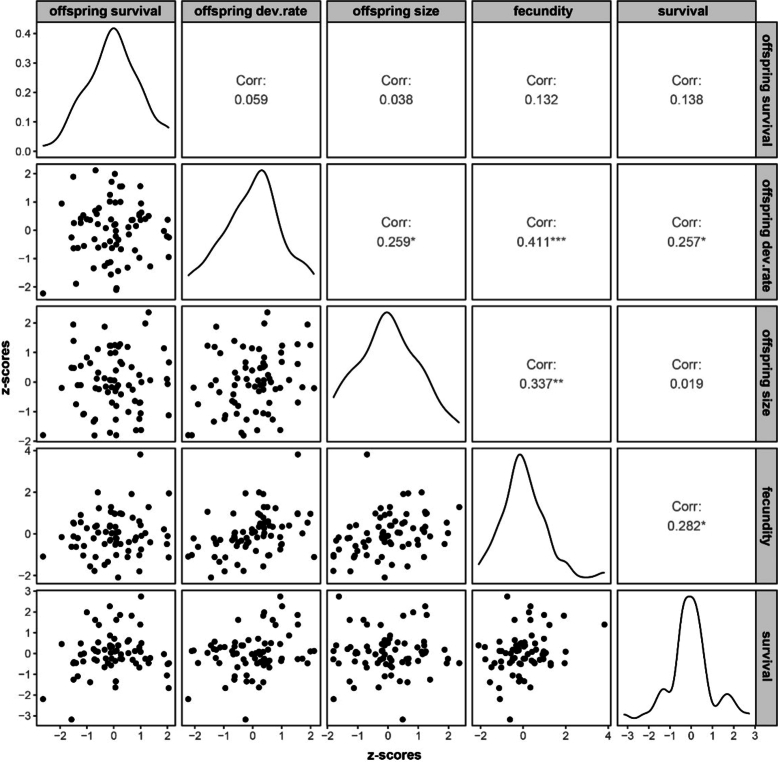
Genetic correlations between fitness components. Data points indicate best linear unbiased predictions (BLUPs). BLUPs for offspring survival, developmental rate, size and adult fecundity were extracted from mixed effect models using restricted maximum likelihood. BLUPs for adult lifespan were extracted from a censored mixed effects cox model. All data have been transformed to z-scores. Source data

The constraint hypothesis would remain viable if most of the quantified mutational variation had deleterious pleiotropic side effects on other unmeasured traits, rendering the variation effectively unusable for adaptive evolution^18^. We tested this hypothesis by quantifying genetic variation in fitness-related traits and wing shape in *S. punctum*. If wing shape evolution is indeed constrained by deleterious pleiotropy, we expect to find genetic covariation between wing shape and fitness components that are functionally unrelated to wing shape or flight. Rearing the 71 isofemale lines of *S. punctum* assayed for wing shape^16^ in a common-garden experiment limiting direct selection on flight (the flies were kept in 50 ml glass vials), we found significant heritable variation among isofemale lines in adult longevity (*χ*^2^_1_ = 8.62, *P* = 0.003), developmental rate (*χ*^2^_1_ = 369.62, *P* < 0.001), juvenile survival (*χ*^2^_1_ = 89.79, *P* < 0.001) and body size (*χ*^2^_1_ = 225.76, *P* < 0.001) but not in early reproductive success (*χ*^2^_1_ = 0.61, *P* = 0.218). About half of all pairwise genetic correlations between fitness components based on best linear unbiased predictors (BLUPs) were positive and statistically significant, indicating that some lines had an overall higher fitness than others. For instance, isofemale lines with high fecundity also had a faster developmental rate (*t*_70_ = 3.74, *r* = 0.41 (95% confidence interval, (0.20, 0.59)), *P* < 0.001), larger adult size (*t*_70_ = 2.97, *r* = 0.34 (0.11, 0.53), *P* = 0.004) and longer adult lifespan (*t*_70_ = 2.44, *r* = 0.28 (0.05, 0.48), *P* = 0.017; Extended Data Fig. 5). This collinearity was also reflected by all five fitness components loading in the same direction on the dominant principal component (PC1) describing trait variation (Fig. 4a). Because individuals with high scores on PC1 had higher fitness across all fitness components and considering that PC1 also explained a larger proportion of the total variation than expected by chance (37.1%; *P*_RAND_ < 0.001; Fig. 4b), these patterns suggest that PC1 captures deleterious pleiotropic alleles affecting life-history traits and variation in overall genetic quality. If wing shape is associated with deleterious side effects, one would thus expect wing shape to covary with PC1. However, PC1 was not related to genetic variation in wing shape (*r*_PLS_ = 0.28, *Z* = 0.14, *P* = 0.445), and we also found no evidence for a relationship between any of the five fitness components and wing shape when all variables were simultaneously analysed in a two-block PLS analysis (Fig. 3c; *r*_PLS_ = 0.39, *Z* = 0.54, *P* = 0.299). To test for stabilizing selection, we also estimated the correlation between the isofemale lines’ fitness and their multivariate residuals from the mean wing shape. None of the correlations between wing shape residuals and the five fitness correlates were significant (Fig. 4d; |*r*| < 0.21, *P* > 0.085). All these analyses were repeated while excluding a single outlier (Extended Data Fig. 5), with the same result. Thus, on the basis of these data, we found no support for the hypothesis that deleterious pleiotropy acts as an evolutionary constraint on wing shape divergence. We note, however, that our experiment would have had limited power to detect more subtle covariation with fitness.

### The most variable wing traits are not the fastest evolving

**Fig. 5:**
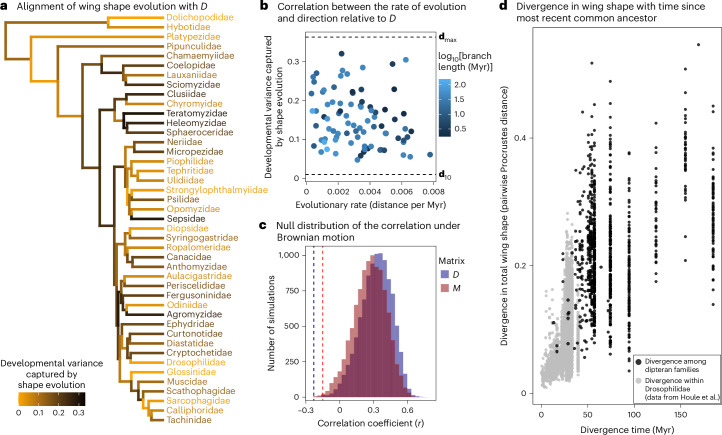
Dimensions with the most developmental variation do not evolve the fastest. **a**, If developmental bias constrains evolution, morphological evolution is expected to be faster if it aligns well with the main axes of the *D* matrix. To test this hypothesis, we first quantified the vector of shape change along the edges of the phylogeny and computed the developmental variance captured by shape evolution. If evolution occurs mainly along the main axes of *D*, this variance will be large and close to the variance explained by the first eigenvector of *D* (**d**_max_), indicating an alignment between shape evolution and developmental bias. **b**, We then correlated the strength of this alignment with the rate of evolution (shape evolution in Procrustes distance per million years) along the branch, expecting a positive relationship if fast rates of evolution are constrained along wing dimensions with high variability. In contrast to this expectation, the rate of evolutionary change does not depend on its alignment with *D* (or *M*; Extended Data Fig. 6). **c**, This observed correlation (dashed vertical lines) is also much lower than what is expected under pure Brownian motion. **d**, The increase in wing shape divergence with phylogenetic distance. The data in grey (below 40 million years) are from Houle et al.^18^. Source data

**Extended Data Fig. 6:**
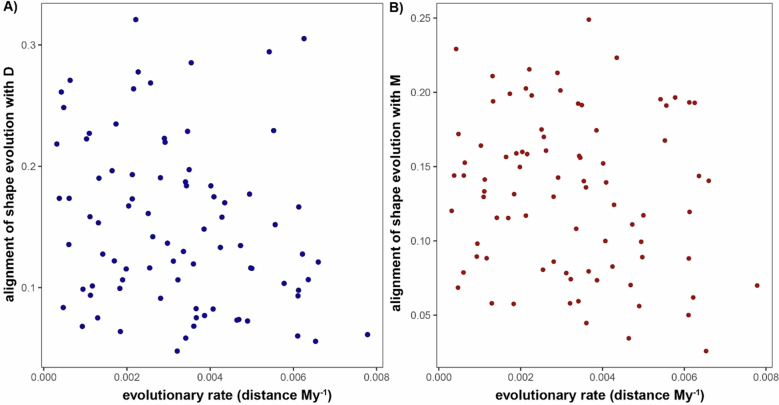
Relationship between evolutionary rate and the amount of developmental variance captured by the vector of evolutionary shape change. **a**,**b**, The relationship between evolutionary rate (in Procrustes distance per My) along individual branches of the phylogeny and the amount of variance in either matrix captured by the vector of evolutionary shape change. In contrast to the expectation based on the constraint hypothesis, the speed of evolutionary change does not increase when its direction aligns with the main axes of either matrix. Source data

If developmental bias indeed acts as a constraint on evolution, we would also predict macroevolutionary divergence along phenotypic dimensions with little developmental variance to be relatively slow^1,2,4^. To test this prediction, we first reconstructed the evolutionary history of wing shape and quantified the alignment between developmental (*D*) or mutational (*M*) bias and the direction of evolutionary shape change on each branch of the phylogeny. Here we quantified these alignments as the proportion of the trace of *D* (or *M*) that was captured by the shape change vector along individual branches. We then tested whether evolutionary shape changes more closely aligned with the main axes of *D* (or *M*) have been faster than wing shape changes along axes with low developmental variability. Contrary to the expectation under the constraint hypothesis, we found no strong correlation between evolutionary rate and the alignment of divergence with *D* (*r* = −0.23) or *M* (*r* = −0.15) (Fig. 5b and Extended Data Fig. 6). The observed correlations are significantly lower than that expected under simulated Brownian motion (both *P*_RAND_ < 0.01; Fig. 5c).

**Extended Data Fig. 7:**
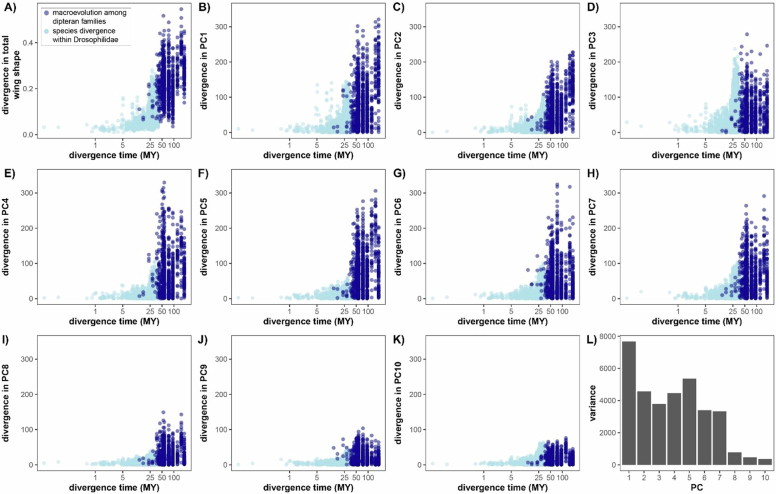
Increase of phenotypic differences with macroevolutionary divergence. Accumulation of divergence in overall wing shape (quantified by Procrustes distance, **A**), as well as the first 10 principal components with evolutionary time (**B-K**). For comparison with the common subspace analysis, the wing shape data were first multiplied by 1,000 and then projected onto the eigenvectors of the phenotypic variance covariance matrix (**P**) estimated in *Sepsis fulgens (*as in the main analyses presented in the main text*)*. Panel **L**) shows the total amount of variance in our dataset along the first ten eigenvectors of **P**. Data on species divergence among drosophilids (spanning up to 40 My) are from^18^. Source data

Together, our results render a simple constraint hypothesis unsuited to explain the observed pattern of macroevolution, which is perhaps unsurprising given the implausibility of genetic drift or unidirectional selection on wing characters over such long timescales. Holstad et al.^19^ recently reported a correlation between evolvability and macroevolutionary rates and argued that such patterns can emerge from rapid fluctuating selection around a global phenotypic adaptive zone defined by persistent stabilizing selection common to all taxa. In this scenario, evolving taxa are constantly tracking, but lagging behind, rapid shifts in phenotypic optima. Such adaptive tracking would be more efficient for traits with abundant genetic variation, resulting in greater differentiation between taxa, whereas traits with low levels of variation would show greater lags and less differentiation, creating a positive correlation between trait evolvability and macroevolution while evolutionary divergence would remain overall low. However, Holstad et al. based their conclusions on patterns observed over a few million years (typical divergence times <1 million years), and, as they point out, the signal of fluctuating selection and associated tracking would probably get overridden by episodes of genetic drift and divergent selection operating over the long macroevolutionary timescales we study here^19,55^ (see also ref. ^56^). Indeed, our data on fly wings show the footprint of substantial accumulated divergence between lineages, tracing far back to deep splits in the dipteran phylogeny (Fig. 5d and Extended Data Fig. 7). Moreover, the fluctuating selection scenario does not by itself generate a strong phylogenetic signal in the data over time. In contrast, the phylogenetic signal in our data explained 86% of the macroevolutionary variance among families (the mean phylogenetic heritability weighted by the total amount of species variance of each shape variable^18^). Our results thus seem incompatible with the constraint hypothesis invoking fluctuating selection around stationary phenotypic optima envisioned by Holstad et al.^19^.

### Correlational selection as a causative factor shaping development and evolution

An alternative explanation for the observed alignments emerges if we consider that different dimensions of the wing may experience different strengths of directional and stabilizing selection, such that developmental bias has itself evolved to align with the fitness surface^6,24,57,58^. Under this scenario, proportionality between *D*, *M* and *R* is observed, not because development constrains macroevolutionary rates, but because pervasive correlational selection has shaped developmental variability, mutational effects and divergence to occur along similar phenotypic dimensions. One such pervasive force is correlational selection for optimal allometric relationships between morphological characters^2,39,59^. Indeed, insect wings show strong allometric scaling, probably due to functional constraints^60,61^. We therefore tested whether the observed patterns could be explained by allometric scaling so that the relationship between *R* and *D*/*M* could solely be ascribed to allometric scaling across Diptera.

**Fig. 6:**
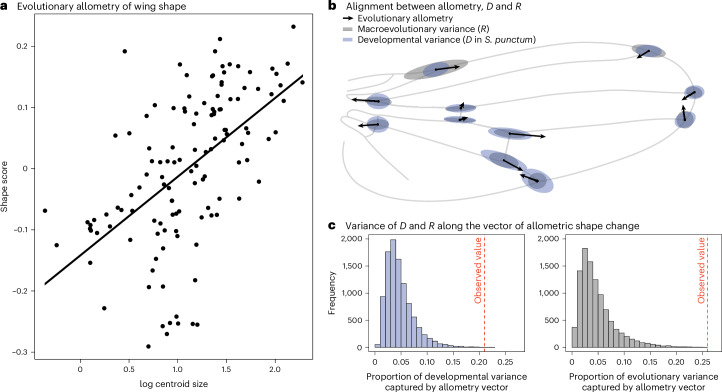
Allometry aligns with both developmental and evolutionary variance. **a**, Wing shape changes with size across 127 species of flies (spanning 30 different families), indicating allometric scaling of shape (multivariate regression of shape against log centroid size: *F*_1,126_ = 18.7, *P* < 0.001, *R*^2^ = 0.13). **b**, These evolutionary allometric shape changes align with the main axes of developmental and macroevolutionary variance in fly wings. **c**, These alignments are much stronger than expected by chance. The displacement of relative landmark positioning with an evolutionary increase in wing size is indicated by black arrows in **b**. Source data

Studying the subset of illustrations and photographs that had an associated scale bar (*n* = 127 species), we found evidence for interspecific allometry (multivariate regression of shape against log centroid size: *F*_1,126_ = 18.7, *P* < 0.001, *R*^2^ = 0.13), which correlates with the intraspecific wing shape allometry vector previously documented in *S. punctum* (vector correlation: *r* = 0.5, *P* < 0.001; ref. ^62^). This is consistent with studies suggesting conserved allometric scaling across Diptera^60^. This interspecific allometric vector captured more variation in both *D* and *R* than expected by chance (*P*_RAND_ < 0.001), indicating that correlational selection for optimal allometric scaling may be causally involved in shaping developmental bias and macroevolutionary divergence in wing shape (Fig. 6a). Interestingly, however, when recalculating the *R* matrix on the basis of residual wing shape after the effect of a common allometric slope was removed, we still recovered a strong alignment between developmental bias and evolutionary divergence (*M*: *β* = 0.71 (0.56, 0.83), *r* = 0.82 (0.68, 0.90); *D*: *β* = 0.55 (0.42, 0.66), *r* = 0.79 (0.69, 0.88)). Hence, while our analysis provides indirect support for a role of correlational selection on allometric scaling in driving the alignment between developmental bias and deep divergence, the alignment persists even when controlling for the effect of allometry.

## Discussion

Here we leveraged within-species estimates of developmental (*D*) and mutational (*M*) variability to assess developmental bias and show that this bias can predict macroevolutionary diversification in deep time. Quantitative genetic theory rests firmly on the assumption that, owing to genetic constraints on rates of evolution, accurate estimates of mutational and genetic covariance matrices can be used to predict evolutionary change in morphological characters. However, much debate remains surrounding the utility of these approaches when applied on longer evolutionary timescales^4,12,14,22,24^.

First, it remains uncertain whether there is enough stability in the amount of standing genetic variation in correlated characters (captured in the genetic variance–covariance matrix, *G*; ref. ^2^) to allow accurate predictions of their long-term evolution^13^. Indeed, if selection and drift reshape *G*, then snapshots of standing genetic variation at any point in time are likely to be poor predictors of evolution, even over only a few hundred generations. In contrast to this notion, developmental bias (*D*, *M* and *G*) in fly wings remains surprisingly conserved across the Drosophilidae and Sepsidae (Fig. 3a,b), clades that diverged from each other around 60 million years ago. Similarly, McGlothlin et al.^63^ showed that *G*, while having evolved across species of *Anolis* lizard, had retained its main dimensionality across >20 million years of species divergence.

Second, however, even if *G* and *M* were to remain constant, predictability relies on the consistency of natural selection, which is likely to fluctuate even in the short term^64,65^. It would thus seem that evolutionary prediction might be limited to special circumstances. Yet, alignments between standing genetic variation within populations and macroevolutionary rates over a couple of millions of years have been observed for morphological features in plants, insects and vertebrates^4,19,20,63,66,67^, notably up to 40 million years in *Anolis* lizards^63^. Here we show that the recently reported correlations for fly wings^16,18^ can extend even longer, with wing shape evolution being predictable—at least to some degree—over 185 million years.

Macroevolution has been suggested to unfold at a slow pace along genetic lines of least resistance delineated by the architecture of the developmental system^68^. While such patterns on their own are compatible with evolutionary constraints, they are hard to reconcile with observations of contemporary adaptation being exceedingly fast^69,70^, questioning the general applicability of genetic constraints as an explanation for evolutionary stasis. Indeed, several analyses have demonstrated that evolutionary stasis in the fossil record may not represent slow rates of evolution, but rather abundant adaptive change in response to fluctuating selection within certain boundaries^19,37,56,71,72^. This scenario is compatible with other observations of stasis in shape evolution in the fossil record despite episodes of strong directional selection^73^ and was recently proposed to explain evolvability–macroevolution relationships on the scale of a couple of million years^19^. However, under this scenario, any macroevolutionary divergence due to drift or directional selection would be expected to erode such a relationship over longer evolutionary timescales. Hence, given the time frame of our study and the strong phylogenetic heritability in our data (see also ref. ^18^), it seems doubtful that the hypothesis can explain the evolvability–rate correlations observed for fly wings^19^.

What remains surprising, then, is the conserved alignment between *D* (or *M*) and the observed divergence in the absence of genetic constraints (Fig. 3c). An alternative explanation for our findings is that the observed patterns reflect the forces of stabilizing correlational selection exercising a similar influence on both developmental architectures and species divergence^16,20,22,63^. To what extent genetic architecture and developmental systems can evolve by natural selection is, however, still a controversial question in need of further theoretical and empirical attention. For example, some recent models highlight that correlational selection can reshape *M* on relatively short time scales in specific scenarios^11,34,35,74,75^, while other models suggest that mutational and developmental biases evolve to align with the forces of correlational selection under fairly restrictive conditions^25,26,33,76^. The main quandaries here are (1) whether correlational selection on epistatic interactions can be strong and variable enough among traits to cause the relatively pronounced mutational and developmental biases often observed in quantitative traits, and (2) whether such genetic architectures can be maintained for long enough to stably align with repeated macroevolutionary adaptations. Theory nevertheless suggests that conditions are particularly permissive when selection acts on multiple correlated characters^77^ and fluctuates predictably between alternative fitness optima^78^, which we argue is probably the case for patterns of selection on wing shape allometry, both within and between dipteran species (Fig. 6). Interestingly, allometries are often referred to as examples of constrained evolution owing to the relatively stable nature of allometric exponents, and wing shape is no exception^79–81^. Yet, both theory and data also highlight that allometric relationships may be outcomes of common forces of correlational selection (for example, for morphology^39^, metabolism^82^, growth^83^ and reproductive investment^84^; but see ref. ^80^ for an alternative interpretation regarding wing shape). Further work is needed to understand and describe correlational selection on allometric scaling in fly wings.

What is the most plausible explanation for the observed correlations between measures of developmental bias, evolvability and macroevolutionary rates? Judging from the recent flurry of comparative studies^16,18–20,29^, the answer seems to depend on the timescale and trait under consideration. For fly wing evolution on these large timescales, there has been little evidence for genetic constraints, and a role for correlational selection simultaneously driving *D*, *G* and *R* seems more plausible. However, explanations invoking genetic constraints or natural selection are not mutually exclusive and might simultaneously contribute to the alignment between developmental bias, evolvability and evolution. Our study highlights the conundrum of explaining how these evolvability–rate relationships can persist over such deep macroevolutionary time (see also refs. ^20,29,39^). To understand the fundamental limits of adaptive morphological evolution, future studies must assess the theoretical plausibility of alternative explanations and identify the relevant timescales on which they apply. If developmental biases are indeed shaped by past forces of natural selection, then contemporary rates of adaptive evolution will depend on whether current selection pressures reflect those of the past, and when they do not, to what extent natural selection restructures the genotype–phenotype map and facilitates adaptation to new trait optima.

## Methods

### Quantifying wing shape and divergence across fly taxa

We focused on the evolution of wing shape within the Eremoneura, a clade within the Brachycera characterized by the presence of three larval instars. This clade is about 185 million years old^42^ and includes the dance and long-legged flies (Empidoidea) as well as the Cyclorrhapha (flies that pupate within the cuticle of the last larval instar (that is, the puparium)^42^). We used the phylogenetic relationships among families proposed by Bayless et al.^47^ as the backbone for our comparative analysis.

To quantify the morphological variation within and between families, we took advantage of illustrations and photographs of fly wings from the taxonomic and systematic literature. An initial dataset was sourced from the *Manuals of Nearctic Diptera* and the *Manuals of Afrotropical Diptera*^44,85^. We focused on those families that are represented in the phylogenetic hypothesis generated by Bayless et al.^47^. Additional photographs and illustrations were collected from a wide range of publications (Supplementary Table 1).

Wing vein reduction has evolved numerous times across the phylogeny and can even be present as intraspecific (genetic) polymorphisms^86,87^. Because homology is difficult to establish in these cases, we were unable to include these species in our analysis, in which we only included observations where the locations of all 11 two-dimensional landmarks used in Rohner and Berger^16^ could be assigned. In total, we collected wing shape data from 827 individuals belonging to 53 families. Using tpsDig2 (ref. ^88^), we manually quantified wing morphology as depicted on the illustrations and images. Additional morphometric data originally collected from images were added for 119 species of drosophilids from Houle et al.^18^ and 36 sepsid species from Rohner and Berger^16^. The final dataset contained 993 observations of 933 species in 530 genera and 68 families. Family affiliations of individual genera were checked using the Systema Dipterorum repository^89^.

The number of observations varied strongly across families (mean, 17.40; median, 12; minimum, 1; maximum, 130). This uneven sampling was caused by a varying number of species per family (for example, Australimyzidae is a monogeneric family containing just 9 described species, whereas Tachinidae has 9,626 species^90^), the loss of landmarks in several species (for example, Sphaeroceridae^87^) and often incomplete illustrations or photographs showing only part of the wing (for example, Muscidae). The landmark coordinates were aligned to the mean configuration of Houle et al.^18^ using Procrustes analysis in MorphoJ (v.1.07a)^91^.

To illustrate the main axes of morphological variation, we applied a canonical variate analysis in the statistical package MASS (v.7.3-55)^92^. This ordination technique finds the axes that maximize variation among families. For the canonical variate analysis, we only considered those families with five or more individual observations.

### Estimating the phylogenetic variance–covariance matrix

The phylogeny includes the placement of individual families. We thus used the observations of different species within these families as repeated measures to approximate the phenotypic variation within the families. To calculate evolutionary rates per million years, we calibrated the phylogeny described previously^47^ using the R package ape (v.5.0)^93^, modelling correlated substitution rate variation among branches. The approximate age of the Eremoneura (185 million years)^42^ and the divergence between Drosophilidae and Muscidae and Tephritidae (estimated to be 29–80 and 48–86 million years, respectively^94^) were used as calibration points. We computed the phylogenetic variance–covariance matrix *R* on the basis of the inverse of the relationship matrix among families (*S*^−1^)^45^ using animal models in ASReml-R. To account for variation due to repeated observations within each family, we added species as an additional random effect (using ‘ide()’ structure). The samples within families thus serve as replicated measures. We only included families for which we had at least five species in our dataset.

### Estimating the dimensionality of covariance matrices

Although all our analysed matrices contain 18 dimensions (due to the loss of four dimensions for scaling, rotation and positioning during Procrustes analysis), geometric morphometric covariance matrices are often rank deficient due to redundant covariance among landmark variables^95^. To assess how many dimensions of *R* had statistical support, we fitted reduced-rank factor analytic mixed models in ASReml-R. We began by fitting a covariance model with a single dimension and continually increased the number of dimensions until increasing the number of dimensions did not lead to a significant increase in model fit (on the basis of Akaike’s information criterion). We then extracted the reduced-rank variance–covariance matrices from these best-fitting models for further analysis using the R package ASExtra4 (v.1.1)^96^. Error variances were estimated separately for each shape variable in all models.

### Comparison of variance–covariance matrices

The *D*, *G* and *P* matrices for sepsids were taken from ref. ^16^. In brief, that study estimated *G* and *P* matrices on the basis of a common-garden experiment with 71 isofemale lines deriving from seven populations of *S. punctum* and 42 lines and nine populations of *S. fulgens*. The *D* matrix was calculated on the basis of 87 male *S. punctum* and 96 male *S. fulgens*. *M*, estimated in its homozygous state in *Drosophila*, was extracted from ref. ^18^. Even though we focused on comparisons between *D* in *S. punctum* and our other variance–covariance matrices of interest, we compared the variances of these matrices along the eigenvectors of *P* estimated in *S. fulgens* to minimize bias in estimates of regression slopes^48^. Following refs. ^48,49^, we decomposed the *P* matrix estimated in *S. fulgens* into its eigenvectors *K*_*D*_ and calculated the variance along *K*_*D*_ for each of the respective variance–covariance matrices of interest as the diagonal entries of the matrix *K*_*D*_^T^*XK*_*D*_, where T denotes transposition and *X* refers to the matrix being compared (*D* and *G* for *S. punctum*; *M* for *D. melanogaster*; *R* for all Diptera measured). We then calculated Pearson’s correlation coefficients (*r*) and OLS slopes (*β*) between these logarithmized variances for a given pair of matrices. To avoid comparing matrices along null spaces with deficient variance, each matrix pair was compared along only the first *k* dimensions of *P*, with *k* equal to the rank of the matrix with the lowest rank (ten dimensions in all cases).

To provide 95% confidence limits around correlations and slopes, we resampled the variance–covariance matrices from the factor analytical models with the best support (on the basis of Akaike’s information criterion), using the REML-MVN approach^97^. This approach uses asymptotic resampling of REML estimates, taking advantage of the fact that the sampling distributions of variance–covariance matrices are well approximated by a multivariate normal distribution at large sample sizes. We performed the MVN resampling on the G-scale using the mvtnorm package for R (see also refs. ^66,67^). With this approach we resampled 10,000 matrices of each kind and subjected them to the common subspace analysis.

### Quantifying genetic quality and deleterious pleiotropy

To quantify fitness variance across the same isofemale lines of *S. punctum* as measured for wing shape, we reared all 71 lines (originating from the seven European populations) in a common-garden experiment including nine temperature treatments ranging from 15 to 31 °C. Note that these isofemale lines were created by pairing a single male and female, expanding the population size to 100–200 flies immediately over a single generation and then maintaining the lines at *n* ≈ 200 for five to ten generations before the experiment. Thus, the studied among-line difference is likely to reflect more dominance variance than expected in a natural population due to inbreeding, but not to any extreme extent due to the rapid population expansion^98^. F_0_ containers with fly cultures were equipped with vials of previously frozen cow dung to attain freshly laid eggs. Each line was seeded with four vials per temperature treatment. For each vial, the juvenile development rate was estimated as the inverse of the time (in days) between the date of a laid clutch and the subsequent emergence of F_1_ adults. Juvenile survival was calculated as the fraction of laid eggs that emerged as adults. Emerged F_1_ females were paired with a male from the same line and placed in a 50 ml vial with access to sugar, water and cow dung as an egg-laying substrate. The sugar, water and dung were replaced every 5 days for the first 15 days. Early reproductive success was estimated as the total number of offspring produced within the first 15 days of adult female life, excluding females that died during this time frame (probably due to accidental deaths). Females that did not lay any eggs during this period were not included in estimates of early reproductive success. Lifespan was estimated as the time from the start of the experiment until the focal female died. A total of 245 females (17%) did not die during the period of observation and were recorded as censored data. After their death, females were measured for their tibia length as an estimate of body size^99^. In total, 1,445 females were measured across all lines and temperature treatments. These females produced a total of 173,556 offspring.

To test for heritable variation in early reproductive success, juvenile survival, developmental rate and body size, we used mixed-effects models using restricted maximum likelihood as implemented in ASReml-R^46^. Temperature treatment, population and their interaction were fitted as fixed effects. Line was added as a random effect. Note that we did not estimate line-by-treatment interactions (that is, *G*-by-*E*) as our aim was to capture overall differences in genetic quality among lines. Repeating the analysis excluding the highest (31 °C) and lowest (15 °C) (that is, the most stressful) temperature treatments led to similar results. Residual variances were allowed to vary across treatments. The significance of the random effect of line was tested using likelihood ratio tests. BLUPs were extracted and used for further analysis. For the analysis of adult lifespan, we fitted a censored mixed-effects Cox model using the coxme package^100^ using treatment and population as fixed effects and line as a random effect. Likelihood ratio tests were used to test whether line effects were significant. BLUPs were extracted by taking the inverse of the hazard ratio for each line as our estimates for adult longevity.

We applied principal component analysis on the correlation matrix based on BLUPs to inspect the loadings on PC1. To test whether the proportion of variance explained by PC1 is larger than expected by chance, we used two different randomization procedures. First, we simulated 10,000 random and unstructured covariance matrices based on the same sample size as in our real data (using the R function rnorm^101^) to generate a null distribution for the proportion of variance explained by PC1. Second, we generated an alternative null distribution by randomizing breeding values among families 10,000 times and calculated the relative eigenvalue of the first eigenvector that was compared with the observed eigenvalue for PC1.

To test for genetic correlations between wing shape and fitness, we used two-block PLS regression as implemented in geomorph 4.0 (ref. ^102^). This is an ordination technique that finds the latent variables within two sets of variables with maximal covariance between the two sets of variates. We used wing shape in the first block. To account for shape allometry and local adaptation, we used the residuals of a multivariate regression of shape on centroid size and population. The second block consisted of scores on PC1 based on the five fitness correlates or breeding values for the five variables separately. Significance was assessed using permutation tests (10,000 random permutations).

### Estimating the relationship between the rate and direction of evolution with respect to *D*

To test whether evolutionary change that aligns with the main axes of *D* is faster than evolutionary change in other directions (as expected if *D* constrains macroevolution), we reconstructed ancestral wing shape at each node and extracted the vectors of shape change observed on each edge of the phylogeny (using the gm.prcomp function in geomorph). We then quantified the developmental variance captured by shape evolution as:

$${e}_{{\mathbf{\upbeta}} }=\,\frac{{{\mathbf{\upbeta}} }^{{\mathrm{T}}}{D}^{\prime }{\mathbf{\upbeta}} }{{\left|{\mathbf{\upbeta}} \right|}^{2}}$$

where **β** is the evolutionary shape change vector of interest, and *D*′ is the *D* matrix scaled by its trace. If evolution occurs primarily along the main axes of *D*, we expect *e*_**β**_ to be large and close to the variance explained by the first eigenvector of *D* (**d**_max_). In contrast, if shape evolution is independent of *D*, this variance is expected to be small and closer to the variance explained by the tenth eigenvector of *D* (**d**_10_). To test for constraints, we computed the correlation between *e*_**β**_ and the magnitude of the shape change along all edges in the phylogeny (measured as shape change in Procrustes distance per million years), expecting a positive correlation if fast rates of evolution are constrained to occur along dimensions with high developmental variability. These analyses were repeated using *M* as the base of comparison.

To compare the observed correlation between the rate of evolution and its direction with respect to *D*, to the expected correlation under Brownian motion, we simulated wing shape evolution using the mvSIM function implemented in the mvMORPH package (v.1.1.7)^103^. To simulate Brownian motion, where the evolutionary changes are more likely to occur along the dimensions of high evolvability, we sampled evolutionary changes from the distribution N(*μ*,*Σ*), where *μ* is a 22-dimensional vector of means equal to zero and *Σ* is a covariance matrix set equal to *D* scaled to the same size as *R*. In these simulations, evolutionary changes are thus governed by Brownian motion but constrained by the orientation of *D*. For each simulation, we then computed *e*_**β**_ as described above and compared the resulting distribution of *e*_**β**_ and its correction with *D* to the observed values. We again repeated this analysis using the *M* matrix.

### Testing for allometry

To assess whether allometric scaling is associated with *D*/*M*, we calculated centroid size for those specimens where scale bars were available (*n* = 127). We then used a multivariate regression of wing shape on log centroid size to estimate the evolutionary allometric shape change vector (using procD.lm in geomorph). A phylogenetic regression of family mean shape on mean centroid size was also significant and resulted in a very similar vector (vector correlation: *r* = 0.92, *P* < 0.001). The alignment between this vector and *D* and *R* was calculated using the method described above. Allometric shape changes were visualized using shape scores following ref. ^104^.

### Reporting summary

Further information on research design is available in the Nature Portfolio Reporting Summary linked to this article.

## Supplementary information

Supplementary Information Supplementary Tables 1 and 2.

Reporting Summary

## Source data

Source Data Fig. 1 PLS scores.

Source Data Fig. 2 Phylogenetic tree file and principal components.

Source Data Fig. 3 Covariance matrices (scaled to unit size for comparison).

Source Data Fig. 4 Morphological and fitness mean values for each isofemale line.

Source Data Fig. 5 Evolutionary rates and phenotypic divergence with time.

Source Data Fig. 6 Shape scores and evolutionary allometry vector.

Source Data Extended Data Fig. 2 Principal components for data sourced from scientific illustrations and photographs.

Source Data Extended Data Fig. 3 Scores generated by the canonical variate analysis.

Source Data Extended Data Fig. 4 Amount of developmental and macroevolutionary variance along all 18 dimensions.

Source Data Extended Data Fig. 5 Scaled mean values for fitness variables.

Source Data Extended Data Fig. 6 Evolutionary rates and alignment with *D*/*M*.

Source Data Extended Data Fig. 7 Divergence in wing shape with increasing phylogenetic distance.

## Figures and Tables

**Extended Data Table 1:**
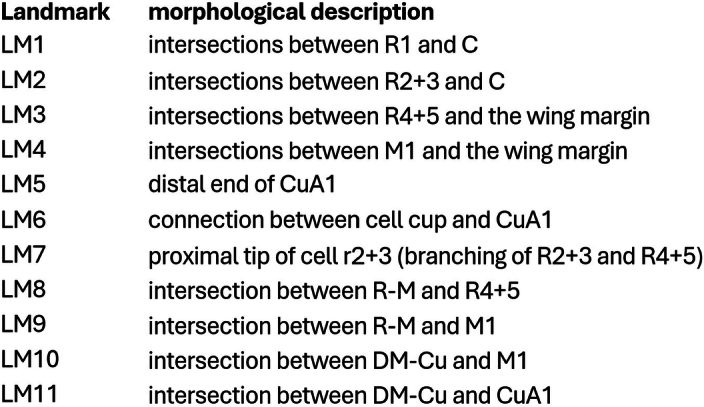
Morphological description of landmarks

Landmarks were defined based on the position of the longitudinal wing veins (Costa (C), Radius (R1-5), Media (M1-3), Cubitus (CuA1)) and the two cross-veins R-M and DM-Cu, as well as the wing cells that are delineated by these veins (see Extended Data Fig. 1). Nomenclature follows 108 (also see^43^).

**Extended Data Table 2:**
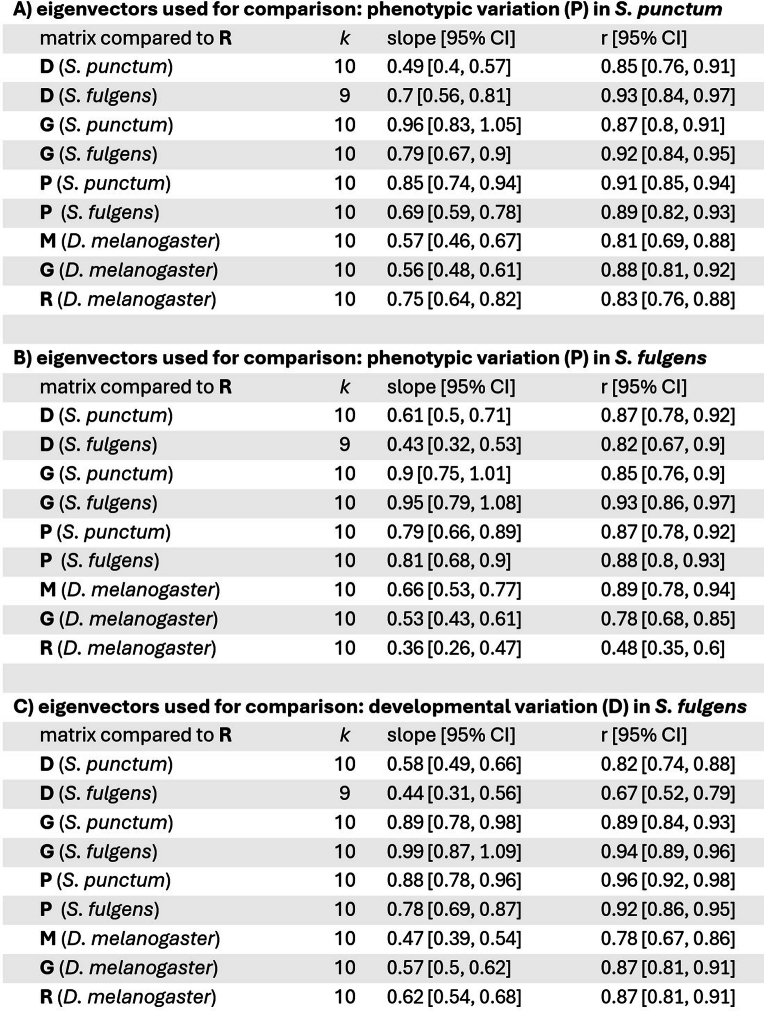
Common Subspace Analysis

A modified version of Krzanowski’s common subspace analysis^18,48,49^ was used to compare different covariance matrices along an arbitrary set of orthogonal phenotypic dimensions (or eigenvectors). Because the choice of reference matrix can bias estimates of slopes and correlation coefficients, all comparisons were repeated using different sets of eigenvectors. *k* refers to the number of dimensions considered for comparisons.

**Extended Data Table 3:**
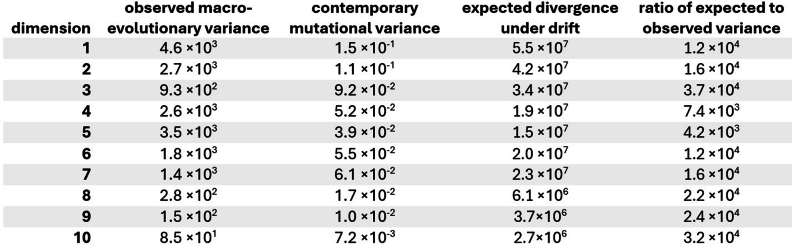
Expected amounts of macroevolution under drift

Observed and expected amount of macroevolutionary variance along 10 phenotypic dimensions under a pure drift scenario. The expected divergence under drift was calculated as two times the mutational variance along each dimension times 1.85 ×10^8^ (this is equivalent to assuming one fly generation per year for 180MY). For consistency with Fig. 3 and S6, the dimensions 1−10 are the first 10 eigenvectors of the phenotypic variance covariance matrix (**P**) estimated in *S. fulgens*. In all instances, the observed macroevolutionary variance is more than 4,000 times smaller than expected under a pure drift scenario. Shape variables were multiplied by 1,000 (as in^18^)

## Data Availability

The wing shape and fitness data are available via Dryad at 10.5061/dryad.08kprr599 (ref. ^105^). Source data are provided with this paper.
