# Supplementary material for: Macroevolution along developmental lines of least resistance in fly wings
Source: Nat Ecol Evol. 2025 Feb 7;9(4):639–51. doi: 10.1038/s41559-025-02639-1 (PMC11976274; doi:10.1038/s41559-025-02639-1)
Supplement: Supplementary file 1 — Supplementary Tables 1 and 2. [file 41559_2025_2639_MOESM1_ESM.pdf]

nature ecology & evolution

Supplementary information

https://doi.org/10.1038/s41559-025-02639-1

Macroevolution along developmental lines of least resistance in fly wings

In the format provided by the authors and unedited

Supplementary Table 1: Sources of illustrations and pictures of fly wings, including total sample sizes (n).

taxon	source	n
Acartophthalmidae	(1-3)	4
Agromyzidae	(1, 4-10)	16
Anthomyzidae	(8, 11-14)	25
Asteiidae	(7, 8, 15)	4
Atelestidae	(16)	1
Aulacigastridae	(17)	15
Australimyzidae	(18)	1
Calliphoridae	(8, 19-26)	14
Canacidae	(7, 8)	8
Carnidae	(7, 8)	3
Chamaemyiidae	(1, 7, 8, 27, 28)	12
Chloropidae	(1, 29)	3
Chyromyidae	(8, 30, 31)	11
Clusiidae	(1, 7, 8)	13
Coelopidae	(1, 7, 8, 31-33)	10
Cryptochetidae	(1, 27, 31, 34-36)	10
Curtonotidae	(1, 7, 37)	17
Diastatidae	(7, 8, 38)	17
Diopsidae	(8, 39-42)	31
Dolichopodidae	(1, 7, 16, 43)	39
Drosophilidae	(1, 8, 27, 44)	130
Empididae	(16, 43, 45)	3
Ephydridae	(1, 8, 46)	12
Fergusoninidae	(28, 41, 47-52)	16
Glossinidae	(53-57)	5
Heleomyzidae	(7, 8, 58-62)	30
Hybotidae	(16, 43)	12
Lauxaniidae	(1, 7, 8)	31
Micropezidae	(8, 41, 63, 64)	12
Muscidae	(8, 65-69)	25
Neriidae	(1, 7, 8, 41, 63, 64, 70-72)	11
Odiniidae	(1, 8, 73-79)	21
Opomyzidae	(7, 8, 80)	28
Paraleucopidae	(8, 81)	2
Periscelididae	(1, 7, 8, 31, 82-87)	16
Piophilidae	(1, 7, 8, 88, 89)	14
Pipunculidae	(1, 7, 8, 27, 53)	15
Platypezidae	(1, 7, 8)	22

Psilidae	(1, 7, 8, 41, 90)	11
Ropalomeridae	(1, 91, 92)	5
Sarcophagidae	(8, 27, 93-99)	20
Scathophagidae	(8, 100-102)	11
Sciomyzidae	(7, 8, 103, 104)	30
Sepsidae	(1, 8, 105-107)	38
Sphaeroceridae	(7, 8, 108)	8
Strongylophthalmyiidae	(41, 109)	6
Syringogastridae	(41, 110, 111)	11
Tachinidae	(8, 53, 112)	17
Tephritidae	(7, 8, 113)	72
Teratomyzidae	(114, 115)	13
Ulidiidae	(1, 7, 8, 113, 116-118)	47

Supplementary Table 2: Leave-one-out cross validation success of the Canonical Variate Analysis (CVA). Only families with 5 or more observations were considered (n = 884).

	**sample size**	**cross validation success**
Agromyzidae	16	0.75
Anthomyzidae	25	0.96
Aulacigastridae	14	1.00
Calliphoridae	13	0.77
Canacidae	8	0.25
Chamaemyiidae	12	0.75
Chyromyidae	11	1.00
Clusiidae	12	0.75
Coelopidae	10	0.80
Cryptochetidae	10	1.00
Curtonotidae	17	0.82
Diastatidae	16	0.81
Diopsidae	31	0.90
Dolichopodidae	39	0.87
Drosophilidae	130	0.92
Ephydridae	11	0.55
Fergusoninidae	16	1.00
Glossinidae	5	1.00
Heleomyzidae	6	0.17
Hybotidae	12	1.00
Lauxaniidae	30	0.60
Micropezidae	11	0.91
Muscidae	22	0.59
Neriidae	11	0.64
Odiniidae	21	0.86
Opomyzidae	27	1.00
Periscelididae	16	0.31
Piophilidae	13	0.38
Pipunculidae	15	1.00
Platypezidae	22	1.00
Psilidae	10	1.00
Ropalomeridae	5	1.00
Sarcophagidae	19	0.68
Scathophagidae	10	0.70
Sciomyzidae	29	0.72
Sepsidae	38	0.97
Sphaeroceridae	8	0.88
Strongylophthalmyiidae	6	1.00
Syringogastridae	11	1.00
Tachinidae	16	0.63
Tephritidae	71	0.97
Teratomyzidae	13	1.00
Ulidiidae	46	0.74

References

1. C. H. Curran, The families and genera of North American Diptera. *The Families and Genera of North American Diptera*. (1934).
2. R. P. la Fuente, C. Hoffeins, J. Rohacek, A new Acartophthalmites Hennig from Eocene Baltic amber (Diptera, Acalyptratae). *Zookeys* 10.3897/zookeys.737.20639, 125-139 (2018).
3. J. Roháček, The second species of Acartophthalmites from Baltic amber (Eocene), with notes on the relationships of the genus (Diptera: Acalyptrata). *Acta Entomologica Musei Nationalis Pragae* **56**, 409-422 (2016).
4. S. Boucher, "Leaf-Miner Flies (Diptera: Agromyzidae)" in Encyclopedia of Entomology*,* J. L. Capinera, Ed. (Springer Netherlands, 2008).
5. F. Hazini, A. A. Zamani, M. Sasakawa, E. Rakhshani, M. Torabi, A Contribution to the Agromyzid Leaf Miners (Diptera: Agromyzidae) of Kermanshah, Iran. *Journal of the Entomological Research Society* **15**, 101-107 (2013).
6. T. Kamiji, R. Iwaizumi, Four Species of Agromyzidae (Dpitera) Intercepted by Japanese Import Plant Quarantine. *Research Bulletin of the Plant Protection Service Japan*, 53-61 (2013).
7. A. H. Kirk-Spriggs, B. J. Sinclair, *Manual of Afrotropical Diptera Volume 3* (South African National Biodiversity Institute, 2021).
8. J. McAlpine *et al.*, *Manual of Nearctic Diptera. Volume 2* (Research Branch, Agriculture Canada, 1987).
9. R. E. Palacios-Torres *et al.*, Identificación, distribución y plantas hospederas de diez especies de Agromyzidae (Insecta: Diptera), de interés agronómico en México. *Acta zoológica mexicana* **24**, 07-32 (2008).
10. F. A. Valenzuela-Escoboza *et al.*, Identificación y fluctuación poblacional del minador de la hoja Liriomyza trifolii en chile jalapeño en el norte de Sinaloa. *Acta zoológica mexicana* **26**, 585-601 (2010).
11. J. Roháček, K. N. Barber, Revision of the New World species of Stiphrosoma Czerny (Diptera: Anthomyzidae). *Beiträge zur Entomologie* **55** (2005).
12. J. Roháček, *A monograph of Palaearctic Anthomyzidae (Diptera)* (Slezské zemské muzeum, 2006).
13. J. Roháček, Anthomyzidae (Diptera) of Taiwan: new species but no new records. *Acta Entomologica Musei Nationalis Pragae* **60**, 269-290 (2020).
14. J. Roháček, New species and records of Anthomyzidae (Diptera) from the East Palaearctic, with a checklist of taxa occurring in the area. *Acta Entomologica Musei Nationalis Pragae* **61**, 261-288 (2021).
15. O. Duda, 58b. Asteiidae. *Die Fliegen der Palearktischen Region* **6** (1934).
16. J. McAlpine *et al.*, *Manual of Nearctic Diptera. Volume 1* (1981).
17. A. Rung, W. N. Mathis, *A Revision of the Genus Aulacigaster Macquart (Diptera: Aulacigastridae)* (Smithsonian Institution Scholarly Press, 2011).
18. I. Brake, W. N. Mathis, Revision of the genus Australimyza Harrison (Diptera : Australimyzidae). *Syst Entomol* **32**, 252-275 (2007).
19. E. B. Dixon, Wing Variations Found in Screw-Worm Flies, Cochliomyia hominivorax, and Three Other Calliphoridae (Diptera). *Annals of the Entomological Society of America* **55**, 210-212 (1962).
20. M. J. Hall, N. MacLeod, A. H. Wardhana, Use of wing morphometrics to identify populations of the Old World screwworm fly, Chrysomya bezziana (Diptera: Calliphoridae): a preliminary study of the utility of museum specimens. *Acta Trop* **138 Suppl**, S49-55 (2014).

21. F. J. Jimenez-Martin, F. J. Cabrero, A. Martinez-Sanchez, Wing morphometrics for identification of forensically important blowflies (Diptera: Calliphoridae) in Iberian Peninsula. *J Forensic Leg Med* **75**, 102048 (2020).
22. K. Limsopatham *et al.*, A molecular, morphological, and physiological comparison of English and German populations of Calliphora vicina (Diptera: Calliphoridae). *PLoS One* **13**, e0207188 (2018).
23. L. Lutz, K. A. Williams, M. H. Villet, M. Ekanem, K. Szpila, Species identification of adult African blowflies (Diptera: Calliphoridae) of forensic importance. *Int J Legal Med* **132**, 831-842 (2018).
24. K. Szpila, A. Zmuda, K. Akbarzadeh, A. Tofilski, Wing measurement can be used to identify European blow flies (Diptera: Calliphoridae) of forensic importance. *Forensic Sci Int* **296**, 1-8 (2019).
25. T. Whitworth, Keys to the genera and species of blow flies (Diptera: Calliphoridae) of America North of Mexico. *Proceedings of the Entomological Society of Washington* **108**, 689-725 (2006).
26. M. Wolff, Y. Ramos-Pastrana, J. R. Pujol-Luz, A new species of Bonatto (Diptera, Calliphoridae, Mesembrinellinae) from Colombia. *Rev Bras Entomol* **57**, 129-132 (2013).
27. C. P. Clausen, *Entomophagous insects* (McGraw-Hill book Company, Incorporated, 1940).
28. J. R. Malloch, Notes on Australian Diptera. No. vi. *Proceedings of the Linnean Society of New South Wales* **50**, 80–97 (1925).
29. P. R. Riccardi, J. W. Ismay, First record of Psilacrum Becker 1912 (Chloropidae, Diptera) from Brazil, with the description of a new species. *Zootaxa* **4329**, 497-500 (2017).
30. M. J. Ebejer, A revision of Afrotropical Chyromyidae (excluding Gymnochiromyia Hendel) (Diptera: Schizophora), with the recognition of two subfamilies and the description of new genera. *African Invertebrates* **50**, 321-434 (2009).
31. E. Stresemann, B. Klausnitzer, *Stresemann - Exkursionsfauna von Deutschland, Band 2: Wirbellose: Insekten* (Spektrum Akademischer Verlag, 2011).
32. L. Cheng, Marine insects. (1976).
33. D. K. Mcalpine, Review of the Australian Kelp Flies (Diptera, Coelopidae). *Syst Entomol* **16**, 29-84 (1991).
34. K. A. Bader, I. J. Al-Jboory, First record of the parasitoid Cryptochetum jorgepastori (Cadahia, 1984)(Cryptochetidae: Diptera) from Jordan. *EPPO Bulletin* **52**, 479-483 (2022).
35. Y. Q. Xi, X. M. Yin, Three new Cryptochetum Rondani, 1875 (Diptera: Cryptochetidae) from Yunnan Province, China and an identification key to Chinese species. *European Journal of Taxonomy* **605**, 1-15 (2020).
36. Y. Q. Xi, Y. L. Guan, X. M. Yin, Rondani, 1875 Newly Recorded from Vietnam, with Descriptions of Three New Species (Diptera: Cryptochetidae). *Annales Zoologici* **70**, 679-686 (2020).
37. A. H. Kirk-Spriggs, A revision of Afrotropical Quasimodo flies (Diptera: Schizophora; Curtonotidae). Part III - the Malagasy species of Curtonotum Macquart, with descriptions of six new species. *African Invertebrates* **52**, 391-456 (2011).
38. D. A. Barraclough, A synopsis of the Afrotropical Diastatidae (Diptera), with the description of five new species from southern and east Africa and first record of the Campichoetinae. *Annals of the Natal Museum* **33**, 13-36 (1992).
39. H. R. Feijen, C. Feijen, A new species of Diopsis L. (Diptera: Diopsidae) from South Africa and Swaziland, and brief review of African species with a large apical wing spot. *African Invertebrates* **53**, 125-142 (2012).

40. H. R. Feijen, C. Feijen, "Diopsidae" in Manual of Afrotropical Diptera. Volume 3. Brachycera—Cyclorrhapha, excluding Calyptratae. Suricata 8. , A. H. Kirk-Spriggs, B. J. Sinclair, Eds. (South African National Biodiversity Institute, Pretoria, 2021).
41. O. Lonsdale, Family groups of Diopsoidea and Nerioidea (Diptera: Schizophora)-Definition, history and relationships. *Zootaxa* **4735**, zootaxa 4735 4731 4731 (2020).
42. G. Ribak, M. L. Pitts, G. S. Wilkinson, J. G. Swallow, Wing shape, wing size, and sexual dimorphism in eye-span in stalk-eyed flies (Diopsidae). *Biol J Linn Soc* **98**, 860-871 (2009).
43. A. H. Kirk-Spriggs, B. J. Sinclair, *Manual of Afrotropical Diptera Volume 2* (South African National Biodiversity Institute, 2017).
44. D. Houle, G. H. Bolstad, K. van der Linde, T. F. Hansen, Mutation predicts 40 million years of fly wing evolution. *Nature* **548**, 447-450 (2017).
45. G. O. Poinar, F. E. Vega, A new genus of Empididae (Diptera) with enlarged postpedicels in mid-Cretaceous Burmese amber. *Historical Biology* **33**, 1830-1835 (2021).
46. E. T. Cresson, Descriptions of new genera and species of the dipterous family Ephydridae. XI. *Transactions of the American Entomological Society (1890-)* **60**, 199-222 (1934).
47. K. Harris, First record of Fergusoninidae (Diptera: Schizophora) outside Australia: a new species of Fergusonina on Syzygium in India. *Syst Entomol* **7**, 211-216 (1982).
48. L. A. Nelson, S. J. Scheffer, D. K. Yeates, Two new species of sympatric Fergusonina Malloch flies (Diptera: Fergusoninidae) from bud galls on high-elevation snow gums (Eucalyptus pauciflora Sieb. ex Spreng. complex) in the Australian Alps. *Australian Journal of Entomology* **50**, 356-364 (2011).
49. L. A. Nelson, S. J. Scheffer, D. K. Yeates, Species diversity of Malloch gall flies (Diptera: Fergusoninidae) forming leaf bud galls on snow gum (Sieb. ex Spreng. complex), with a description of a new species from Tasmania. *Insect Systematics & Evolution* **43**, 147-160 (2012).
50. G. S. Taylor, K. A. Davies, The gall fly, Fergusonina lockharti Tonnoir (Diptera: Fergusoninidae) and description of its associated nematode, Fergusobia brittenae sp. nov. (Tylenchida: Neotylenchidae). *Journal of Natural History* **44**, 927-957 (2010).
51. G. S. Taylor, Revision of Fergusonina Malloch gall flies (Diptera : Fergusoninidae) from Melaleuca (Myrtaceae). *Invertebr Syst* **18**, 251-290 (2004).
52. G. Taylor, K. Davies, N. Martin, T. Crosby, First record of Fergusonina (Diptera: Fergusoninidae) and associated Fergusobia (Tylenchida: Neotylenchidae) forming galls on Metrosideros (Myrtaceae) from New Zealand. *Syst Entomol* **32**, 548-557 (2007).
53. M. Buck *et al.*, Key to Diptera families-adults. *Manual of Central American Diptera* **1**, 95-156 (2009).
54. C. J. De Beer *et al.*, Using genetic and phenetic markers to assess population isolation within the southernmost tsetse fly belt in Africa. *Onderstepoort Journal of Veterinary Research* **86**, 1-8 (2019).
55. J. Hargrove *et al.*, Wing length and host location in tsetse (Glossina spp.): implications for control using stationary baits. *Parasit Vectors* **12**, 24 (2019).
56. D. Kaba *et al.*, Phenetic and genetic structure of tsetse fly populations (Glossina palpalis palpalis) in southern Ivory Coast. *Parasit Vectors* **5**, 153 (2012).
57. C. P. Klingenberg, G. S. McIntyre, Geometric Morphometrics of Developmental Instability: Analyzing Patterns of Fluctuating Asymmetry with Procrustes Methods. *Evolution* **52**, 1363-1375 (1998).
58. S. Y. Mun, S. J. Suh, Taxonomic revision of the genus Suillia Robineau-Desvoidy (Diptera: Heleomyzidae) from Korea. *Journal of Asia-Pacific Biodiversity* **12**, 400-406 (2019).

59. J. Preisler, M. Tkoc, Two new species of Heleomyzidae (Diptera) from Czech Republic and Crimea. *Acta Entomologica Musei Nationalis Pragae* **58**, 267-274 (2018).
60. A. J. Woznica, A. H. Kirk-Spriggs, "Diopsidae" in Manual of Afrotropical Diptera. Volume 3. Brachycera—Cyclorrhapha, excluding Calyptratae. Suricata 8. *,* A. H. Kirk-Spriggs, B. J. Sinclair, Eds. (South African National Biodiversity Institute, Pretoria, 2021).
61. A. J. Woznica, New records of the Polish Heleomyzidae (Diptera) with taxonomic notes on the Central European fauna. *Annals of the Upper Silesian Museum* **4**, 179-192 (1993).
62. A. J. Woznica, A new species of the genus <i>Suillia</i> Robineau-Desvoidy, 1830 from Cameroon and Kenya (Diptera: Heleomyzidae). *African Invertebrates* **53**, 1-5 (2012).
63. M. L. Aczel, Diptera: Neriidae and Micropezidae (Tylidae) *Insects of Micronesia* **14**, 47-90 (1959).
64. E. T. Cresson, The Neriidae and Micropezidae of America north of Mexico (Diptera). *Transactions of the American Entomological Society (1890-)* **64**, 293-366 (1938).
65. V. M. Alves, M. O. Moura, C. J. B. de Carvalho, Wing shape is influenced by environmental variability in Polietina orbitalis (Stein) (Diptera: Muscidae). *Rev Bras Entomol* **60**, 150-156 (2016).
66. V. Michelsen, Costal vein chaetotaxy, a neglected character source in Fanniidae and Muscidae (Diptera: Calyptratae). *European Journal of Taxonomy* **826**, 94-134 (2022).
67. S. S. Nihei, C. J. B. De Carvalho, The Muscini flies of the world (Diptera, Muscidae): identification key and generic diagnoses. *Zootaxa*, 1-24 (2009).
68. A. Pereira-Colavite, C. J. B. De Carvalho, Taxonomy of Neomuscina Townsend (Diptera, Muscidae) from Brazil. *Zootaxa* **3504**, 1-55 (2012).
69. A. C. Pont, Studies on Australian Muscidae (Diptera) II. A revision of the tribe Dichaetomyiini Emden. *Bulletin of the British Museum (Natural History) Entomology* **23**, 191-286 (1969).
70. D. A. Barraclough, The southern African species of Neriidae (Diptera). *Annals of the Natal Museum* **34**, 1-17 (1993).
71. E. R. Chame-Vazquez, G. Sanchez-Hernandez, M. D. Estrada-Marroquin, Contribución al conocimiento de la familia Neriidae (Diptera) en Chiapas, México: nuevos registros y uso potencial de la morfometría geométrica. *Revista de la Sociedad Entomológica Argentina* **81** (2022).
72. T. A. Sepúlveda, C. J. B. de Carvalho, A. Pereira-Colavite, Systematics of the Neotropical genus Loxozus (Diptera: Neriidae), with notes on distribution and sexual dimorphism. *Zoologia-Curitiba* **36**, 1-6 (2019).
73. S. D. Gaimari, Order Diptera, Family Odiniidae. *Fauna of the United Arab Emirates* **4**, 780-783 (2011).
74. S. D. Gaimari, "Odiniidae" in Manual of Afrotropical Diptera. Volume 3. Brachycera—Cyclorrhapha, excluding Calyptratae. Suricata 8. *,* A. H. Kirk-Spriggs, B. J. Sinclair, Eds. (South African National Biodiversity Institute, Pretoria, 2021).
75. V. Hernandez-Ortiz, J. F. Dzul-Cauich, A new species of Neotraginops Prado (Diptera: Odiniidae) from Mexico and Belize, with additional records for Odinia coronata Sabrosky in Mesoamerica. *Zootaxa* **3786**, 593-599 (2014).
76. D. E. O. F. Limeira, D. W. A. Marques, G. A. Reis, J. A. Rafael, Inpauema, a new genus of Odiniidae (Diptera) from Brazil, with description of five new species. *Zootaxa* **4362**, 517-534 (2017).
77. D. E. O. F. Limeira, D. W. A. Marques, S. D. Gaimari, J. A. Rafael, Pauximyia, a new genus of Odiniidae (Diptera: Acalyptratae) with description of two new species from Brazil. *Zootaxa* **4728**, zootaxa 4728 4722 4724 (2020).

78. M. Parchami-Araghi *et al.*, First Iranian record of the family Odiniidae (Diptera: Opomyzoidea), including two species new to the Middle East region. *Zootaxa* **4471**, 580-584 (2018).
79. A. Trres, J. A. Rafael, S. D. Gaimari, D. E. O. F. Limeira, Revision of the genus Lopesiodinia Prado, 1973 (Diptera: Odiniidae) with description of three new species, and a key to the extant Neotropical genera and species of Traginopinae. *Zootaxa* **5052**, 332-352 (2021).
80. C. M. Drake, A review of the British Opomyzidae (Diptera). *British Journal of Entomology and Natural History* **6**, 159-176 (1993).
81. T. A. Wheeler, B. J. Sinclair, Systematics of Paraleucopis Malloch with proposal of Paraleucopidae, a new family of acalyptrate Diptera. *Zootaxa* **4668**, zootaxa 4668 4663 4661 (2019).
82. R. Ale-Rocha, G. Freitas, W. N. Mathis, Revision of the Neotropical genus Marbenia Malloch (Diptera: Periscelididae). *Zootaxa* **3872**, 365-375 (2014).
83. G. Freitas, R. Ale-Rocha, Description of two new species of Stenomicra Coquillett (Diptera, Periscelididae) from the Neotropics. *Rev Bras Entomol* **55**, 348-354 (2011).
84. D. A. Grimaldi, The Asteioinea of Fiji (Insecta: Diptera: Periscelididae, Asteiidae, Xenasteiidae). *American Museum Novitates* **2009**, 1-59 (2009).
85. W. N. Mathis, A. Rung, Redescription of the genus Diopsosoma Malloch (Diptera, Periscelididae). *Rev Bras Entomol* **48**, 303-309 (2004).
86. L. Pollini Paltrinieri, J. Roháček, Periscelis (Myodris) haennii sp. nov., a new species of Periscelididae (Diptera) from Ticino, Switzerland, with a new key to European species of the subgenus. *Alpine Entomology* **6**, 39-49 (2022).
87. A. Rung, R. Ale-Rocha, New species of Cyamops (Diptera: Opomyzoidea: Periscelidae) from the old and new world tropics. *Zoologia-Curitiba* **28**, 803-811 (2011).
88. J. Lopez-Garcia, C. Angell, D. Martin-Vega, Wing morphometrics for the identification of Nearctic and Palaearctic Piophilidae (Diptera) of forensic relevance. *Forensic Sci Int* **309**, 110192 (2020).
89. D. MartÍN-Vega, A. Baz, V. Michelsen, Back from the dead:Thyreophora cynophila(Panzer, 1798) (Diptera: Piophilidae) 'globally extinct' fugitive in Spain. *Syst Entomol* **35**, 607-613 (2010).
90. M. Buck, S. A. Marshall, The identity of Pseudopsila, description of a new subgenus of Psila, and redefinition of Psila sensu lato (Diptera : Psilidae). *Eur J Entomol* **103**, 183-192 (2006).
91. R. Ale-Rocha, M. Pollet, First records of Ropalomeridae (Diptera, Acalyptratae) from French Guiana. *Zoosystema* **41**, 1-5 (2019).
92. S. Ibáñez-Bernal, V. Hernández-Ortiz, The new genus Acrocephalomyia, and a new species of Ropalomera from Costa Rica, with additional records for other Mesoamerican species (Diptera: Ropalomeridae). *Zootaxa* **3478**, 553-569 (2012).
93. H. Kurahashi, C. Samerjai, Revised keys to the flesh flies of Thailand, with the establishment of a new genus (Diptera: Sarcophagidae). *Medical Entomology and Zoology*, 67-93. (2018).
94. T. Pape, Two new species of Phylloteles Loew from Namibia (Diptera: Sarcophagidae). *Journal of the Entomological Society of Southern Africa* **48**, 273-276 (1985).
95. G. Pekbey, A new species of Sarcophaga (Pandelleisca) (Diptera, Sarcophagidae) from Turkey. *Zookeys* **937**, 129-138 (2020).
96. N. Sontigun *et al.*, Wing morphometric analysis of forensically important flesh flies (Diptera: Sarcophagidae) in Thailand. *Acta Trop* **190**, 312-319 (2019).

97. K. Szpila, N. P. Johnston, K. Akbarzadeh, R. Richet, A. Tofilski, Wing measurements are a possible tool for the identification of European forensically important Sarcophagidae. *Forensic Sci Int* **340**, 111451 (2022).
98. Y. G. Verves, L. A. Khrokalo, Review of the genus Sphenometopa Townsend, 1908 (Diptera: Sarcophagidae) of the Middle East. *Biologia* **75**, 1643-1656 (2020).
99. M. Zhang, W. W. Chu, T. Pape, D. Zhang, Taxonomic review of the Sphecapatodes ornata group (Diptera: Sarcophagidae: Miltogramminae), with description of one new species. *Zoological Studies* **53**, 48 (2014).
100. A. L. Ozerov, M. G. Krivosheina, A review of the genus Scathophaga Meigen, 1803 (Diptera: Scathophagidae) of Russia. *Russian Entomological Journal* **30**, 201-246 (2021).
101. A. L. Ozerov, A review of the genus Pogonota Zetterstedt, 1860 (Diptera: Scathophagidae) in Russia. *Russian Entomological Journal* **25**, 185-207 (2016).
102. M. A. Schäfer *et al.*, Geographic clines in wing morphology relate to biogeographic history in New World but not Old World populations of dung flies. *Evolution* **(in press)** (2018).
103. F. Kazerani, A. A. Talebi, J. Mortelmans, Taxonomic study of the marsh flies (Diptera: Sciomyzidae) in Iran. *Journal of Insect Biodiversity and Systematics* **3**, 105-117 (2017).
104. L. Knutson, S. Manguin, R. Orth, A second australian species of pherbellia robineaudesvoidy (diptera: sciomyzidae) 1. *Australian journal of entomology* **29**, 281-286 (1990).
105. P. T. Rohner, W. U. Blanckenhorn, A Comparative Study of the Role of Sex-Specific Condition Dependence in the Evolution of Sexually Dimorphic Traits. *Am Nat* **192**, E202-E215 (2018).
106. P. T. Rohner, D. Berger, Developmental bias predicts 60 million years of wing shape evolution. *Proc Natl Acad Sci U S A* **120**, e2211210120 (2023).
107. P. T. Rohner *et al.*, Distribution, diversity gradients and Rapoport's elevational rule in the black scavenger flies of the Swiss Alps (Diptera: Sepsidae). *Insect Conserv Diver* **8**, 367-376 (2015).
108. G. C. Steyskal, A new species of the genus Archiborborus Duda from Mexico (Diptera: Sphaeroceridae). *Journal of the Kansas Entomological Society*, 154-157 (1973).
109. M. Iwasa, N. L. Evenhuis, The Strongylophthalmyiidae (Diptera) of Papua New Guinea, with descriptions of five new species and a world checklist. *Entomological Science* **17**, 96-105 (2014).
110. S. A. Marshall, M. Buck, J. H. Skevington, D. Grimaldi, A revision of the family Syringogastridae (Diptera: Diopsoidea). *Zootaxa* **1996**, 1-80 (2009).
111. J. A. Rafael, J. T. Câmara, M. J. A. Holanda, A new species of Syringogastridae (Diptera, Acalyptratae) from the Amazon Basin and new records for Brazil. *Zootaxa* **3014**, 26-34 (2011).
112. P. Sehnal, (1998). A new species of Borgmeiermyia Townsend, 1935, from Paraguay (Insecta: Diptera: Tachinidae). *Annalen des Naturhistorischen Museums in Wien. Serie B für Botanik und Zoologie*, 349-354 (1998).
113. D. Kovac, E. Kameneva, V. Korneyev, A review of Tephritidae and Ulidiidae (Diptera, Tephritoidea) of Croatia. *Zoodiversity* **56** (2022).
114. D. K. Mcalpine, R. G. Dekeyzer, Generic Classification of the Fern Flies (Diptera, Teratomyzidae) with a Larval Description. *Syst Entomol* **19**, 305-326 (1994).
115. L. Papp, Oriental Teratomyzidae (Diptera: Schizophora). *Zootaxa* **2916**, 1-34 (2011).
116. M. S. El-Hawagry, The family Ulidiidae in Egypt (Diptera: Tephritoidea). *African Entomology* **29**, 445-462 (2021).

117. S. V. Korneyev, M. Hauser, E. P. Kameneva, S. D. Gaimari, A key to species of the Euxesta sororcula species group (Diptera: Ulidiidae: Lipsanini), with new synonymy and a new record from the USA. *Pan-Pacific Entomologist* **98**, 150-162 (2022).
118. A. C. O. Vasconcelos, L. D. Wendt, C. J. B. de Carvalho, Phylogenetic relationships of picture-winged flies of the tribe Pterocallini (Diptera: Ulidiidae) inferred from a morphological analysis. *Austral Entomology* **60**, 330-350 (2021).
